# Supplementary material for: BMP7 Increases UCP1-Dependent and Independent Thermogenesis with a Unique Gene Expression Program in Human Neck Area Derived Adipocytes
Source: Pharmaceuticals (Basel). 2021 Oct 25;14(11):1078. doi: 10.3390/ph14111078 (PMC8625022; doi:10.3390/ph14111078)
Supplement: Supplementary file 1 [file pharmaceuticals-14-01078-s001.zip › pharmaceuticals-1422349-supplementary.pdf]

SUPPLEMENTARY MATERIALS

BMP7 increases UCP1-dependent and independent thermogenesis with a unique gene expression program in human neck area derived adipocytes

Abhirup Shaw[1,2], Beáta B Tóth[1], Rini Arianti[1,2], István Csomós[3], Szilárd Póliska[4], Attila Vámos[1,2], Zsolt Bacso[3,5], Ferenc Győry[6], László Fésüs[1,*,†], Endre Kristóf [1,*,†]

* Authors to whom correspondence should be addressed
† These authors have contributed equally to this work and share last authorship

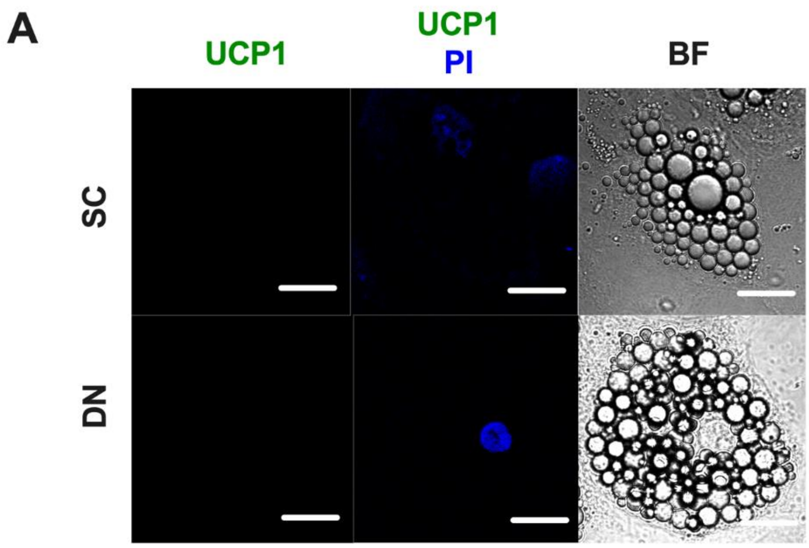

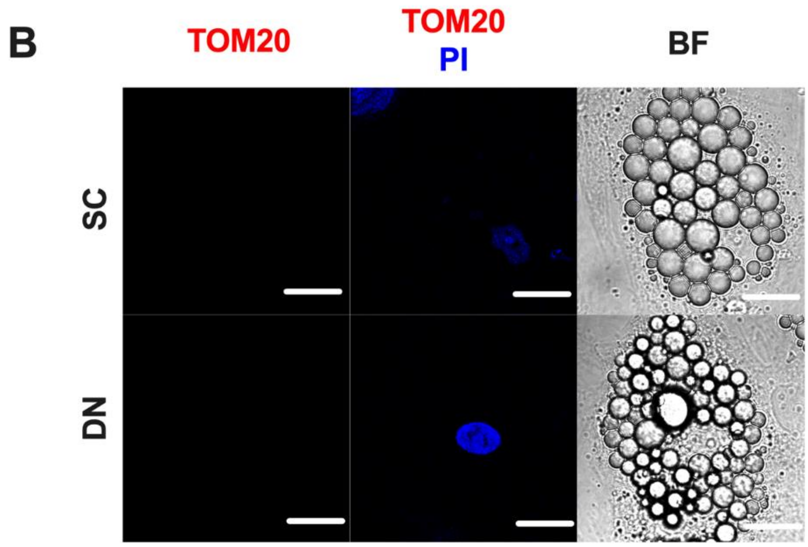

Figure S1: Secondary antibody control images illustrating the specificity of the antibodies used for (A) UCP1 and (B) TOM20 immunostaining.

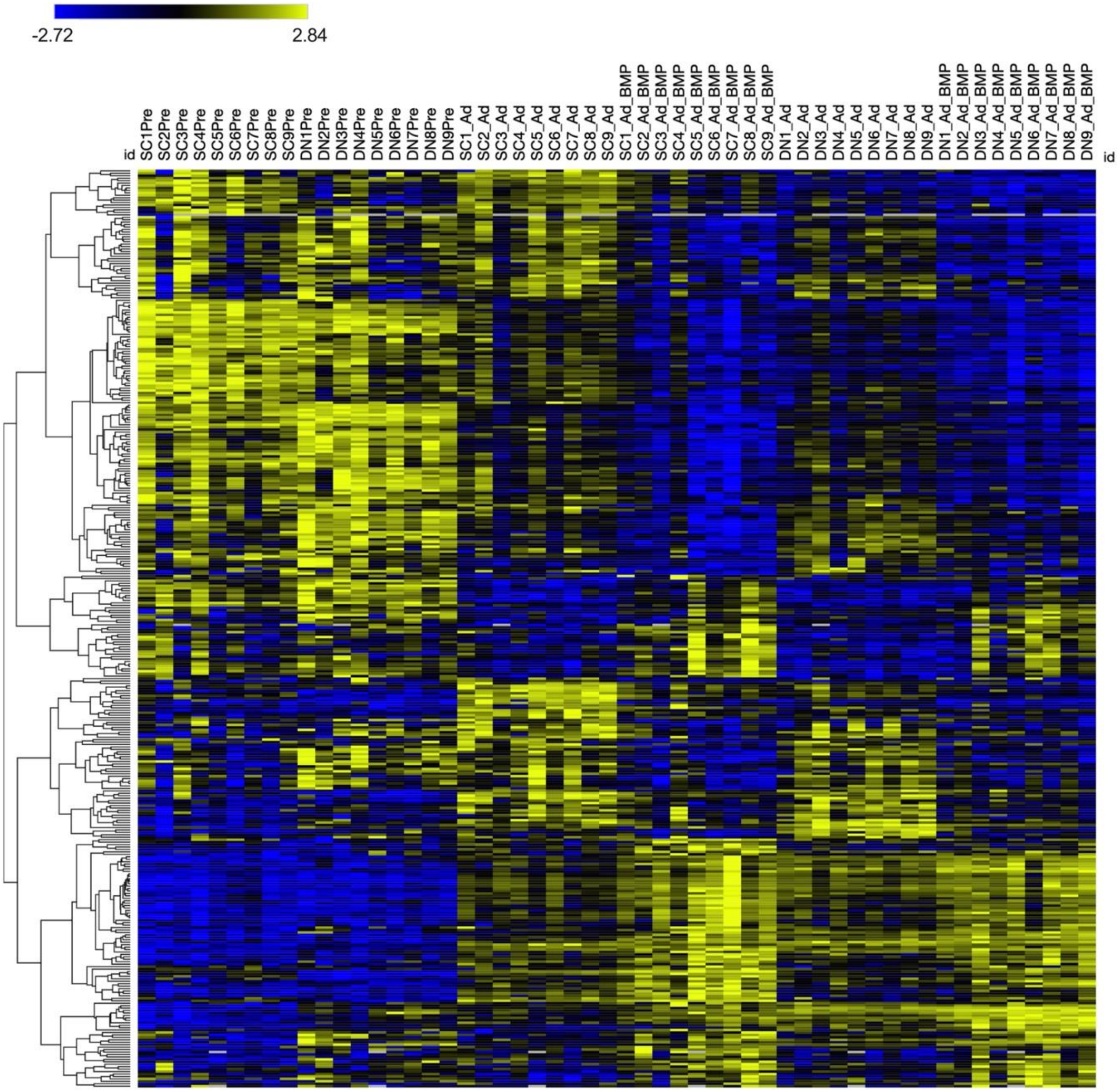

Figure S2: Heatmap illustrating the up- and downregulated genes upon BMP7 treatment in Subcutaneous (SC) and Deep-neck (DN) derived differentiated adipocytes.

Table S1: List of genes upregulated by BMP7 treatment in SC and DN derived differentiated adipocytes arranged in descending order of their log_2fold change.

SC BMP7 Upregulated		SC BMP7 Upregulated	
**Gene symbol**	**log2FoldChange**	**Gene symbol**	**log2FoldChange**
ID1	4.323910086	PTGDR	2.413106326
MYOZ1	3.815257814	AL645608.2	2.376186706
SAMD11	3.784625054	PLN	2.321543994
DES	3.554691177	CNR1	2.199495778
CPNE5	3.309507889	CFAP221	2.169532573
ACTC1	3.306069719	REM1	2.113782599
ACAN	3.296401223	ANGPT4	2.105206616
DLX3	3.284339325	NOG	2.104441062
LINC02600	3.186418672	CADM3-AS1	2.099192777
ID3	3.16046663	HPGD	2.080751198
COMP	3.108907299	CDH22	2.051153334
ADRA2C	3.102683197	COL26A1	1.960028377
CP	2.987441884	SNAI1	1.953656146
SCGN	2.819776974	RELN	1.951226724
LINC02593	2.813611147	CDH20	1.93715909
SLC7A10	2.810247254	SLC38A11	1.931584893
PKD2L1	2.698658775	OCA2	1.921927258
AL513523.1	2.683539568	SMAD6	1.888147012
RSPO2	2.626777174	AF131216.3	1.830018607
CADM3	2.57260893	CD1D	1.822472003
SOX8	2.560843947	FGF13	1.814904133
ACKR1	2.524605317	COL9A3	1.809843487
ATOH8	2.524303159	FNDC11	1.802770928
PARM1	2.466669411	AC026469.1	1.796702366
SOX18	2.41466829	HAAO	1.789572018

SC BMP7 Upregulated		SC BMP7 Upregulated	
**Gene symbol**	**log2FoldChange**	**Gene symbol**	**log2FoldChange**
CHRM4	1.775738385	SYT17	1.391717843
COL9A2	1.747867605	ITGA8	1.367114342
SMAD9	1.745770338	NMUR1	1.335581269
FCN2	1.739685418	REEP1	1.32984759
NPY1R	1.73663662	CDK18	1.326750733
HSPB1P2	1.713445425	FAM47E	1.31893393
HS3ST2	1.71204828	NKD1	1.309467618
CYP26B1	1.709335816	MYL3	1.297578592
AC005077.4	1.689437421	NPAS1	1.285051089
GOLGA7B	1.682268174	PLPPR4	1.266329546
TMEM132C	1.676207435	DMTN	1.260387229
THBD	1.651241299	CRYAB	1.215425559
CABCOCO1	1.631222348	TMEM132B	1.203168431
RPL13AP25	1.628126529	LGI4	1.202562633
SNCG	1.605034763	PTH1R	1.200250783
GLDN	1.592887743	C1QTNF1	1.190330752
ADRA2A	1.574820332	SHISAL1	1.159709646
ONECUT2	1.573457918	LDB3	1.154500762
KLHL23	1.544446163	TICRR	1.151449431
DNAH9	1.465560789	KAZALD1	1.144952849
A2M	1.462757238	SEZ6L2	1.142609772
STOX1	1.446388329	PDZD7	1.126157135
GRIK5	1.444671473	AL590999.1	1.119495944
MYOM1	1.44004003	ITGA9	1.116605064
SCN5A	1.41721036	PRUNE2	1.11027983

SC BMP7 Upregulated	
**Gene symbol**	**log2FoldChange**
CMYA5	1.10122402
SMAD7	1.097474924
EDNRA	1.096823565
SULT1A1	1.091532477
GPD1L	1.080335373
SOX5	1.076249272
AC115837.1	1.066296754
RIMS4	1.059059672
MOCS1	1.044596893
INAFM2	1.044446854
LEPR	1.028497206
HPD	1.021361988
FZD1	1.006403861
KIF7	0.976720615
IL17RE	0.97165896
STBD1	0.971575003
FZD5	0.965670843
SLC6A8	0.952924339
MATN2	0.943991767
PHOSPHO2	0.85854136
HVCN1	0.85470841

DN BMP7 Upregulated		DN BMP7 Upregulated	
**Gene symbol**	**log2FoldChange**	**Gene symbol**	**log2FoldChange**
ID1	3.852371514	PGF	1.909710316
ACAN	3.825115247	STOX1	1.89968918
SAMD11	3.648406106	NOG	1.887943886
CPNE5	3.625372971	MYOZ3	1.874786067
SAMD5	3.546433107	CRTAC1	1.853633218
ACTC1	3.489450739	CD1D	1.852039921
MYOZ1	2.997059593	SMAD9	1.786026121
ADRA2C	2.994785149	COL9A2	1.777219922
AL645608.2	2.818377137	ONECUT2	1.746618002
NPTX2	2.691138574	CFAP221	1.709276352
SOX8	2.546407379	GDAP1L1	1.616393176
LINC02593	2.544341894	SYNDIG1	1.606137257
SOX18	2.518180935	TMEM132B	1.558069466
MDFI	2.500496827	IL7R	1.550378741
CABCOCO1	2.275778027	PTH1R	1.461952975
RELN	2.264223218	PLEKHA6	1.45512684
ID3	2.213056568	TRIM67	1.41517565
CDH22	2.119287966	SNAI1	1.391137702
PARM1	2.113251223	SMAD6	1.378591534
CADM3	2.091992884	TNMD	1.371976192
AL022068.1	2.068941949	TSPAN18	1.327019458
CNTN4	2.044472392	MYOM1	1.324118815
CD22	1.998432045	PCA3	1.279184349
AC009041.2	1.991269877	GLDN	1.251295819
ATOH8	1.979697576	KAZALD1	1.238911603

DN BMP7 Upregulated	
**Gene symbol**	**log2FoldChange**
CRYAB	1.176575401
PRUNE2	1.142913434
SPAG1	1.076299203
EDNRA	1.067123926
MYL3	1.062148163
TICRR	1.049910116
RSPO3	1.029570587
FOXD2AS1	0.97823264
CETN2	0.884590041
SMAD7	0.863214307

Table S2: List of genes downregulated by BMP7 treatment in SC and DN derived differentiated adipocytes arranged in descending order of their log_2 fold change.

SC BMP7 Downregulated		SC BMP7 Downregulated	
**Gene symbol**	**log2FoldChange**	**Gene symbol**	**log2FoldChange**
COL6A6	3.492598001	MIR503	2.132037459
AC008063.1	3.395447666	POU2F2	2.098299248
AC010980.2	3.393805097	SCUBE2	2.059040929
MMP27	3.365143551	HAPLN1	2.058441387
MGAT4C	3.290440027	OPCML	2.016274436
CCL11	3.223681768	AL445250.1	2.010415934
LINC01028	2.715741231	MYHAS	2.00652321
AC010980.1	2.663757613	RSPO1	1.990965817
AIF1L	2.613541683	CD7	1.990728456
FAM180B	2.601043	SERTAD4	1.978229641
MATN4	2.582574024	EGFL6	1.977413285
LRP2	2.576293807	GSG1	1.958498871
DIRAS2	2.574534603	CMKLR1	1.948419303
AC018647.1	2.529676599	ST8SIA1	1.914572209
TMEM176B	2.485714913	SERTAD4-AS1	1.909501918
CDX1	2.44127292	AC007255.1	1.908843047
TRPV6	2.426649136	ALDH1A2	1.893278597
AL356417.2	2.373358662	HPSE	1.848835985
IL17RD	2.309930939	AL139241.1	1.847513323
NEFL	2.257939732	GDF6	1.833806607
PDGFRL	2.234942035	FGF9	1.820369607
TMEM176A	2.228927856	SEC14L5	1.80114166
RASSF2	2.184806244	EGR2	1.795349726
GRIA1	2.156591089	ADRA1D	1.790661712
MGARP	2.132385989	SECTM1	1.786365175

SC BMP7 Downregulated		SC BMP7 Downregulated	
**Gene symbol**	**log2FoldChange**	**Gene symbol**	**log2FoldChange**
USP44	1.782517116	FAM13C	1.548476144
DKK2	1.768970473	PDPN	1.539868423
RERG	1.759079315	ADGRD1	1.53665604
LINC01315	1.743941954	SERPINE2	1.528127098
MYH2	1.740411988	C8orf34	1.52359127
WNT2	1.720179133	AC012349.1	1.522795067
TEK	1.71728211	NRK	1.51960871
ADAMTSL2	1.67852318	AGTR1	1.513481091
AL157996.1	1.67478568	AC018529.2	1.506802093
PMAIP1	1.669144648	CASS4	1.50048109
NCKAP5	1.667808053	B3GALT2	1.497015896
MIR503HG	1.662657223	FRMPD4	1.487272459
SDR42E1	1.66200204	MKX	1.483539158
PPL	1.656094329	LSP1	1.481196263
FAM43B	1.651376685	CCNA1	1.476693836
PRR33	1.621072089	ZNF385D	1.472025328
AC022467.1	1.61973712	RAB38	1.461304851
DPT	1.614864765	THSD1	1.440659482
CELSR1	1.609169497	DPP4	1.438620878
TMC2	1.593461368	FAM198B	1.425253992
ABCA9	1.573855278	MBP	1.410634038
TEX26-AS1	1.566531029	ADAM33	1.384887554
GALNT16	1.560408531	IL20RA	1.383746697
PLP1	1.553806468	PRR26	1.363793991
STXBP6	1.550325341	SNCAIP	1.351481188

SC BMP7 Downregulated		SC BMP7 Downregulated	
**Gene symbol**	**log2FoldChange**	**Gene symbol**	**log2FoldChange**
PRR15	1.350927051	CERKL	1.173015461
EPHB6	1.334804628	GDPD1	1.172678511
AL136295.5	1.332403423	WNT5A	1.167587419
FAM184B	1.321226888	FAP	1.164392699
IL16	1.306482167	GJA1	1.157144154
OSR1	1.305568131	CMAHP	1.15650099
SLIT2	1.29318911	MFAP2	1.155753361
PSTPIP1	1.286946337	PLA2G5	1.15008327
RDH10-AS1	1.276915028	CRABP2	1.146188867
LSP1P5	1.274213406	SLC9A7	1.144515958
KIT	1.269527859	AC144831.1	1.142320922
CREB5	1.259393882	LRRC7	1.135727545
AC080038.3	1.25552875	ARL4C	1.133498553
LINC00968	1.25416263	GFRA1	1.119817829
MFAP4	1.247900452	TFAP2A	1.107661172
GPM6B	1.247617054	MTMR9LP	1.098842219
OLFML2A	1.229743333	WNT5B	1.098514953
NAALAD2	1.226522798	VDR	1.093528169
SLC38A5	1.221650448	FAM198B-AS1	1.092310643
ITGA4	1.194540546	TMEM158	1.083531423
CACNB4	1.194303214	AC130371.2	1.08030199
AC026250.1	1.194120355	RDH10	1.079872936
C17orf58	1.194086309	THSD4	1.073541926
STK32B	1.191687664	LINC01119	1.070725656
WNT5A-AS1	1.186253316	MRPS31P4	1.070312962

SC BMP7 Downregulated		SC BMP7 Downregulated	
Gene symbol	log2FoldChange	Gene symbol	log2FoldChange
NTN4	1.05675178	TBC1D3L	0.935139248
FMNL1	1.043872451	ARHGAP29	0.933359635
GPSM2	1.041373361	FOXP2	0.927914649
ABCA8	1.032425338	ANPEP	0.927465051
FLRT2	1.029462967	CTSC	0.922239143
TMEM204	1.029457625	AC018653.3	0.921377145
TMEM171	1.021542017	GAS6-DT	0.910307509
AC005736.1	1.021397876	AP006623.1	0.892598563
ADGRL4	1.015073832	NID1	0.888837389
PCSK5	0.995732353	TMPO	0.886643059
MAP3K7CL	0.995495108	SQOR	0.876971514
AHNAK2	0.994197205	PLEKHF1	0.871483899
AC092376.2	0.991689988	GRHL1	0.870890942
LURAP1L	0.985990519	COL18A1	0.869476682
RHOJ	0.983428827	LOX	0.864428007
CCND2-AS1	0.978591055		
KCND2	0.964507197		
RNF152	0.963477122		
SGCD	0.961041367		
TUFT1	0.96045794		
SLFN11	0.95336928		
SGK1	0.953339628		
HLX	0.953165359		
PLSCR4	0.937394657		
AC020763.4	0.935333905		

DN BMP7 Downregulated		DN BMP7 Downregulated	
**Gene symbol**	**log2FoldChange**	**Gene symbol**	**log2FoldChange**
KRT1	28.63892582	TNC	1.915520305
MYH8	4.500451579	STXBP6	1.905318302
CCL11	3.104184645	DPT	1.852147588
MATN4	2.965810428	ROBO4	1.84464629
FAM180B	2.799118237	RASSF2	1.794205009
LINC01028	2.734135895	PCSK6	1.792494571
KCNJ16	2.688828127	RSPO1	1.740281089
PI15	2.601137268	CLSTN2	1.707635086
SLPI	2.531668916	SERTAD4-AS1	1.700213394
AIF1L	2.489104446	AL157996.1	1.679154626
MYH1	2.463447047	TRIL	1.648007654
MYH2	2.400310775	MFAP4	1.590473708
AC018647.1	2.39699463	EPSTI1	1.573626545
TMEM176A	2.342278715	IFITM1	1.572409691
AC010976.2	2.208136917	GSG1	1.53123357
SMOC2	2.196500521	CGNL1	1.529673132
TMEM176B	2.162634236	AC079336.5	1.515514372
TEK	2.1607686	ASPN	1.511988943
IL18	2.12921794	NEURL1B	1.480047292
HERC6	2.121904272	KLHL33	1.450837596
TNFSF10	2.081786583	SCUBE2	1.450811159
RERG	2.073546592	COL8A1	1.433485329
MX2	1.986044859	MMP23B	1.416746179
SEC14L5	1.980894274	GALNT16	1.400176036
VEGFD	1.960159058	ABCA9	1.393583438

DN BMP7 Downregulated		DN BMP7 Downregulated	
**Gene symbol**	**log2FoldChange**	**Gene symbol**	**log2FoldChange**
MGARP	1.386587463	ABCA8	1.002609607
IFIT1	1.354564684	DDX58	0.974199842
LTBP2	1.353278269	FAM198B	0.963978957
WISP1	1.305793197	MTMR9LP	0.947131159
CREB5	1.299717209	AXL	0.938696967
ADGRD1	1.292436126	GPSM2	0.935946352
INMT	1.270869263	SLC9A7	0.921206876
AC080038.1	1.243932797	MEG3	0.905092758
TMEM158	1.227349552	RHOJ	0.877572695
FAM198A	1.223417303	KCNK2	0.870507227
FAP	1.211834207	SLFN11	0.85352153
CMAHP	1.186002215	GAS6	0.850090985
MFAP2	1.173100564		
ADAM33	1.155835283		
FLRT2	1.149730166		
C17orf58	1.145423928		
AC144831.1	1.102888609		
MEG9	1.094841605		
SLC1A7	1.083633241		
VDR	1.078555822		
HADHAP2	1.077796216		
CACNB4	1.068996088		
XAF1	1.052361682		
RDH10-AS1	1.017613242		
NID1	1.014749045		

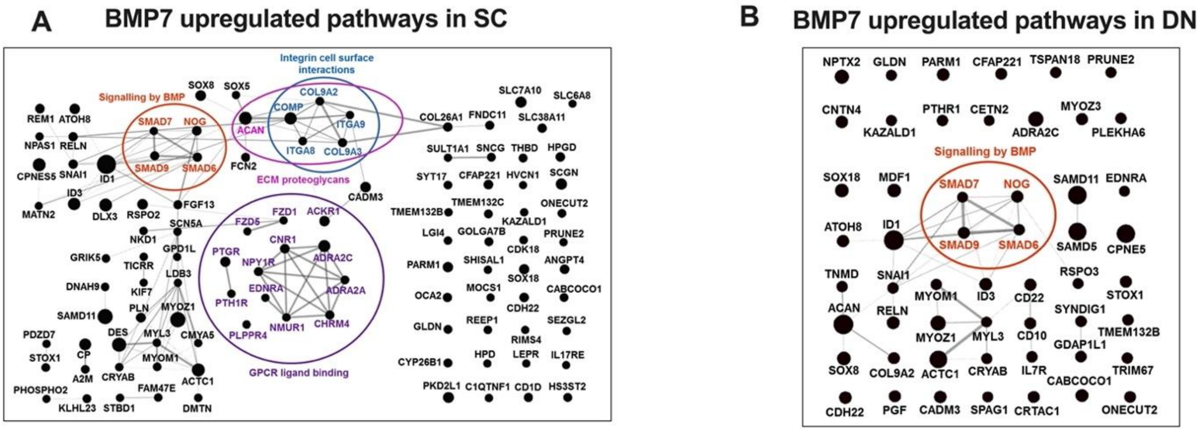

Figure S3: Gephi diagrams illustrating pathways and gene interaction networks upregulated by BMP7 treatment in SC and DN derived differentiated adipocytes.

Table S3: Table listing gene expression assays used in the study

Genes	Assay ID
ID1	Hs00357821_g1
CKMT2	Hs00176502_m1
GAPDH	Hs99999905_m1
UCP1	Hs00222453_m1
ACAN	Hs00153936_m1
CRYAB	Hs00157107_m1
